# Progress in Medicine

**Published:** 1894-10-20

**Authors:** 


### Progress in Medicine.

### INFECTIVE DISEASES.

**Bubonic Plague.**—The outbreak of the Bubonic plague at Hong Kong revives the memory of several historic epidemics so terrible that men feared while writing that none might be left to read what they wrote of it. Keser has recently argued that it formed the Great Plague at Athens in 430, and a new account of the dreadful visitation in 1348 has been compiled by the learned Benedictine, F. A. Gasquet. Twenty-five millions of people are said to have died in Europe during that outbreak, and from a third to half of the English population. We happen to have singularly full accounts of it from English and Italian writers, and the political and social history of England has been ever since affected by the vast changes it caused. The terrors of our Plague year, 1665, have since recurred occasionally in Asia and Eastern Europe. Pringle narrated the course of an outbreak in British Ghurwal in 1864, which appeared to be eradicated by sanitary measures. It was prevalent there again in 1886 and 1888,[1] and known as the Maha Mari, according to Thompson. For some years it has existed in districts of the Chinese mainland, and spread to Hong Kong in April. The usual symptons were noted, and the bacilli were found in the buboes by Yersin and Kitasato, and specimens sent to Europe. They are described by Sims Woodhead,[2] when stained with gentian or fuchsin, as almost like diplococci and enclosed in a delicate capsule, which, however, may be due to the preparation, or as a short bacillus with the ends somewhat rounded. There is a clear band or space in the centre, and their size varies accordiug to the method of staining employed. The results of medical treatment of the patients, or of injection or excision of the buboes, were almost nil according to Cantlie.[3] The epidemic died out after about sixteen weeks' duration, and some 7,000 deaths, but the figures are very uncertain.

**Leprosy.**—The isolation of lepers is being carried out in various English colonies. Some 36 are to be found in the New South Wales Hospital,[4] and a new commission has been formed in South Africa to decide on segregation and treatment.[5] Ten cases are reported from Eastern Prussia.[6] In Norway 960 were known at the end of 1890. In the 35 previous years 7,635 were enumerated, and 186 of these recovered.[7] Nevins Hyde gives an elaborate paper with a full account of the literature of the subject on leprosy in North America. The United States do not seem a favourable soil for the disease, in spite of Norwegian importations.[8] About 600 cases were found sixteen years ago, but their number appears to have decreased greatly, while Cuba alone has three or four hundred. A case of the anæsthetic variety at Birmingham[9] in a man who had lived twenty years in India is described by Douglas Heath, who notices the great sensory and very slight motor disturbances. Professor S. Martins[10] has diagnosed a case of syringomyelia due to leprosy which was verified post-mortem. The cavity in the cervical cord contained large numbers of Hansen's bacilli, thus corroborating the views of Zambaco as to the pathology of leprosy. An elaborate report on the disease in Iceland is contributed by Ehlers.[11] About fifty cases only are reported from there now, and all or nearly all are of the tuberous form, but the statistics are insufficient, and Ehlers believes that more anæsthetic cases could be discovered.

**Cholera.**—Max Gruber contributes to the *Lancet* a long paper on the bacteriological diagnosis of cholera. He concludes from his researches—1. That Koch's views are true, and that there are present in cholera vibrios which are distinguishable from all others found in the intestines, and that they are the cause or a part of the cause of the disease. 2. That the true vibrios occur in several sub-species morphologically different. 3. The microscopical appearances of quite young colonies of Koch's vibrio in 10 per cent. gelatine are almost absolutely characteristic, but many other tests are untrustworthy. Among these are the nitroso-indol reaction, the inoculation of guinea-pigs, and agar, potato, and broth cultivations. 4. There is little difficulty in diagnosing the vibrios found actually in the intestines, but much uncertainty as to those found in water and elsewhere. The flagella of true vibrios are certainly not constant in number, and they vary in other ways. As to 3, no other vibrio (except Dencke's cheese one) gives the irregularly shaped granular and furrowed colony on the surface of a gelatine plate. He reviews the evidence that the true vibrio occurs constantly in true cholera, and not in the intestines of individuals who are free from it, and decides in favour of Koch's view, though they may be found in immune persons living among cholera patients.[12]

Hueppe discusses the question of the cholera poison which he believes to be a derivative of albumen closely allied to albumose, and while Pfeiffer thinks that the bacilli are poisonous in themselves, he insists that the poison is elaborated by them, for dead bacteria produce far less effect than living ones, and protection is afforded by other bacteria, and even by ferments against the bacillus, but not against the cholera poison. This secreted poison can be shown to be the cause of all the symptoms; whether it is of a specific nature is not definitely decided.[13] Natural immunity against cholera is created by the intestinal epithelium, the cells of which contain free acid nuclein. This nuclein takes up the cholera poison and neutralises its toxic action, while setting free the protective part of it. Klemperer[14] believes this work is carried on as long as the nuclein is kept in an acid state, and the nucleolar acid if injected renders animals immune. De Vestea, of Pisa, denied at Rome that there was a general poisoning by nitrites[15]; but pointed out that the comma bacillus not only reduced nitrates, but also separated an acid from carbohydrates. Now nitrates in an acid medium are more active, and here they probably effect a necrosis of the epithelium with local vaso motor paralysis. The varying form of cholera bacilli is the subject of a paper by Metchnikoff,[16] who succeeded in transforming the short and long types into each other and obtained more or less permanent varieties from them. Dempster [17] gave an interesting account of certain experiments to the Medical Chirurgical Society showing that cholera vibrios only survived in moist soil and not in dry. In peaty soil, too, they were quickly killed. All the soils used were sterilised, and therefore no light was thrown on the question of the survival of the cholera among saprophytes and other germs. Confirmation of his results was given by some Indian authorities present, but it would seem that a definite range of moisture, temperature, and other factors are of influence in epidemics.

Haffkine's[18] protective inoculations excite extreme interest in India. In a village of 200 inhabitants 116 were inoculated and ten cases of cholera occurred all among the unprotected, and a great number of persons are being inoculated in various parts of the country. Haffkine uses two vaccines; the first mild form produces some pain, discomfort, and fever for one day. After five days a stronger vaccine is employed, which produces a similar result if the patient has undergone the first stage. He has vaccinated about 25,000 persons since his arrival in India, and in a number of instances cholera has attacked unprotected members of families, while those who were vaccinated escaped.

Duka[19] read a paper at Buda Pest on the disease, in which he mentioned some of the difficulties felt in regarding it as water-borne, such as the infection of villages far away from rivers, and single sporadic cases in barrack rooms. The rapidity with which it travels is also hard to explain. He showed evidence, too, that the Hardwar fair was not the source of the epidemic of 1892, as had been reported. Friedrich investigated[20] the life of the bacillus in contact with various articles of food and drink. Lying on fruits and vegetables it survived from one to five days. The juice of currants, grapes, and some other fruits killed it in an hour or two, wine and cider in from five to twenty minutes, beer in three hours. Draer[21] tested the disinfectant power of sozoiodol preparations and found that the acid and mercury compounds have a very powerful action on the bacillus. Cranberry juice appears to be an excellent means of relieving thirst and vomiting. Goriansky[22] tested it in fifty cases and reports highly of it when ordinary means failed. As he and Friedrich showed, it has a powerful action on the bacillus itself. Musk, which seemed to give some relief in plague cases at Hong Kong, was found of great value in cholera by Monsiorski if given in large doses.[23] It is curious that in the Greenwich epidemic of English cholera last year tobacco exercised a remarkable protective influence upon those who consumed it.[24] The question of the conveyance of cholera by compressed rags in bales was examined at the Dresden Conference, and no evidence could be found that any case had ever occurred, though the loose soiled linen of travellers may undoubtedly carry it.[25] External gangrene in cholera[26] has been only recorded in about twelve cases, according to Martin-Durr. Dry gangrene is twice as frequent as moist. The latter he compares with diabetic gangrene. The former may be due to arterial spasm, arteritis or embolus from endocarditis. The lesions in the kidneys in cholera nostras were described by Bernhardt[27] at the Society of Physicians, Vienna, as precisely similar to those found in Asiatic cholera, with the exception of the coagulation necrosis sometimes seen in the latter. Thus there are fatty degeneration in the convoluted tubercles and hyaline casts, while the glomeruli are intact. He regarded the lesions as due entirely to the loss of water, but Baginsky and Leyden thought that the toxins might have some share in it.

The greatest effort of this year against cholera has been the precautions agreed upon at the International Conference held in Paris. The convention regulates[28] the pilgrim ships from India and Oceania, and the sanitary and police rules under which they shall be; it provides for the watching and care of pilgrims in the Red Sea, and for the protection and sanitary rules of the places of traffic in the Persian Gulf. The carrying out of the regulations are to be entrusted to a commission, sitting in Constantinople, to which very great powers are committed. The internal sanitation of Mecca itself is left for the present to the management of the Sultan, to whom strong representations have been made.

[1] B. M. J., Sept. 8, 1894. [2] Ditto, Aug. 18, 1894. [3] Ditto, Aug. 25, 1894. [4] Ditto, June 9, 1894. [5] Ditto, March 10, 1894. [6] Ditto, May 19, 1894. [7] Ditto, March 10, 1894. [8] Am. Journ. Med. Sci., Sept., 1894. [9] Lancet, June 23, 1894. [10] Med. Week, Ap. 13, 1894. [11] B. J. Dermat, Ap., 94. [12] Lancet July 7. [13] Berlin Klin. Woch., Nov. 17 and 18, 1894. [14] Med. Week, Ap. 27. [15] Lancet, Ap. 14, 1894. [16] B. M. J., Aug. 4, 1894. [17] Ditto, May 26, 1894. [18] Ditto, Aug. 4, 1894. [19] Lancet, Sept. 8, 1894. [20] Am. Journ. Med. Sci., March, 1894. [21] Ditto. [22] B. M. J., July 7, 1894. [23] Prov. Med. Journ., Feb., 1894. [24] N. Y. Med. Journ., Feb. 10, 1894. [25] Am. Journ. Med. Sci., March, 1894. [26] Med. Chron., March, 1894. [27] Med. Week, Feb. 23, 1894. [28] B. M. J., Ap. 28, 1894.

